# Alteration in gut microbiota associated with hepatitis B and non-hepatitis virus related hepatocellular carcinoma

**DOI:** 10.1186/s13099-018-0281-6

**Published:** 2019-01-18

**Authors:** Qisha Liu, Fan Li, Yaoyao Zhuang, Jian Xu, Jianwei Wang, Xuhua Mao, Yewei Zhang, Xingyin Liu

**Affiliations:** 1 Department of Microbiology, Key Laboratory of Pathogen of Jiangsu Province, Nanjing Medical University, Nanjing, China; 2 Key Laboratory of Human Functional Genomics of Jiangsu Province, Nanjing Medical University, Nanjing, China; 3 Key Laboratory of Holistic Integrative Enterology, Second Affiliated Hospital of Nanjing Medical University, Nanjing, China; 4 Department of Hepatobiliary and Pancreatic Surgery, Jiangsu Cancer Hospital, Jiangsu Institute of Cancer Research, Nanjing Medical University Affiliated Cancer Hospital, Nanjing, China; 5 Department of General Surgery, Second Affiliated Hospital of Nanjing Medical University, Nanjing, China; 6 Department of Clinical Laboratory, Affiliated Yixing Hospital of Jiangsu University, Yixing, China; 7 Department of Hepatobiliary and Pancreatic Surgery, Zhongda Hospital, Medical School, Southeast University, Nanjing, China

**Keywords:** Gut microbiome, Dysbiosis, Liver cancer, Hepatocellular carcinoma, HBV

## Abstract

**Background:**

The onset of hepatocellular carcinoma (HCC) ranked fifth malignancies all over the world. Increasing evidences showed that the distribution of HCC was related to the incidence of chronic hepatitis B virus (HBV) infection and other factors, such as alcoholism, aflatoxin B1 ingestion and obesity. Recent studies demonstrated that gut dysbiosis plays an important role in liver diseases. However, the researches on gut microbiota of HBV and non-HBV non-HCV related HCC have not been reported. In this study, we investigated the differences between the gut microbiota of HBV related HCC (B-HCC) and non-HBV non-HCV related HCC (NBNC-HCC), finally found some potential bacteria, linking different pathological mechanism of both types of HCCs.

**Results:**

We carried out 16S rRNA analyses in a cohort of 33 healthy controls, 35 individuals with HBV related HCC (B-HCC) and 22 individuals with non-HBV non-HCV (NBNC) related HCC (NBNC-HCC). We found that the species richness of fecal microbiota of B-HCC patients was much higher than other two groups. Interestingly, the feces of NBNC-HCC patients harbored more potential pro-inflammatory bacteria (*Escherichia*-*Shigella*, *Enterococcus*) and reduced levels of *Faecalibacterium*, *Ruminococcus*, *Ruminoclostridium* which results in decrease potential of anti-inflammatory short-chain fatty acids. The feces of NBNC-HCC patients had relatively fewer abundance of multiple biological pathways related to amino acid and glucose metabolism, but high level of transport and secretion in some types. However, the B-HCC patients had opposite results of bacterial composition and associated multiple biological pathways versus NBNC-HCC patients. Meanwhile, we found that aberrant network of gut microbiota occurred differently in B-HCC and NBNC-HCC patients.

**Conclusions:**

Our study indicated that B-HCC and NBNC-HCC patients showed differential abundance of bacteria involved in different functions or biological pathways. We suggested the modification of specific gut microbiota may provide the therapeutic benefit for B-HCC and NBNC-HCC.

**Electronic supplementary material:**

The online version of this article (10.1186/s13099-018-0281-6) contains supplementary material, which is available to authorized users.

## Background

Hepatocellular carcinoma (HCC) is the most common form of liver cancer being diagnosed annually [1], which is the fifth global malignancy [2, 3]. With increasing the risk factors such as obesity and fatty liver diseases, the worldwide incidence of HCC also increases [3, 4].

The worldwide distribution of HCC was related to the infection of chronic hepatitis B virus (HBV), especially in China. HBV promote carcinogenesis not only through the direct activation of oncogenic proteins but also indirectly establish chronic inflammation, fibrosis and cirrhosis [5]. Approximately 257 million people are infected with HBV worldwide [6]. For patients with HBV induced cirrhosis, 70–90% of HCC occur against a background of cirrhosis [7]. In addition to HBV, other risk factors include alcoholism, aflatoxin B1 ingestion, obesity, age, diet habits and genetics. In this study, we defined the other elements as non-HBV non-HCV related HCC.

The gut microbiota plays a vital role in physiology, nutrition, immunology and pathogenic processes [8]. The liver interacts with the gut through hepatic portal and bile secretion systems. Increasing evidences have revealed that gut microbiota plays an important role in liver disease formation, pathogenesis and treatment responses [9, 10]. It was reported that microbial translocation, bacteria peptidoglycan and metabolic outcomes can intensify the clinical features of chronic liver diseases [11].

Increasing studies have demonstrated that gut dysbiosis affect liver diseases, such as nonalcoholic alcohol-related liver disease, primary sclerosing cholangitis, fatty liver disease (NAFLD), fibrosis, cirrhosis and HCC [8, 12–16]. Most of these diseases presented a potentially “dysfunctional” gut microbiome. For instance, some cirrhotic patients’ gut microbiota had the high abundance of *Enterobacteriaceae*, suggesting it an invasion microbiota from the mouth. Meanwhile, some beneficial bacteria declined in the gut of liver diseases, such as *Lachnospiraceae* [14, 17]. In a recent comprehensive study of gut microbiome in early HCC patients, species diversity decreased in healthy controls compare to cirrhotic patients but increased in early HCC compared with cirrhosis [18]. In addition, butyrate-producing genera declined, however, LPS-producing genera enriched in early HCC patients [18].

However, there are limited comparison researches on gut microbiota of HBV related HCC and non-HBV non-HCV related HCC. The aim of this study was to find the differences in the gut microbiota composition of HBV and non-HBV non-HCV related HCC compared with healthy controls using 16S rRNA sequencing. This would be helpful to find the potential bacteria linking different pathological mechanisms between HBV and non-HBV non-HCV related HCC. It also helps to develop a new non-invasive differential diagnosis and therapeutic procedures for HCC patients with harboring specific gut bacteria.

## Results

### Cohorts of patients

Volunteers information was collected, including age, gender, weight, height, drinking history, body mass index (BMI) (kg/m^2^) and blood biochemical indexes (Table 1, Additional file 1). Only two HCC patients were assessed as the Child–Pugh class B, other HCC patients were Child–Pugh class A (Additional file 1). The average MELD score of HCC patients was five (Additional file 1). In total, 2047 operational taxonomic units (OTUs) were obtained from fecal microbiota of three groups for healthy controls, HBV related HCC (B-HCC) patients and non-HBV non-HCV (NBNC) related HCC (NBNC-HCC) patients, averaging 1749, 1285 and 1696, respectively. As shown in Table 1, the coverage values were nearly 1.00 for the sequences in three groups, which indicated that the sequencing depth was enough for the investigation of fecal microbiota of HCC patients or healthy controls.

**Table 1:** The basic information of primary data analysis, and species richness indices in the fecal samples

Characteristic	Healthy controls	NBNC-HCC patients	B-HCC patients
Patients	33	22	35
Gender (male/female)	11/22	18/4	28/7
Median age	56.64 ± 9.91	61.12 ± 9.99	56.83 ± 9.95
Drinking condition	22/5/3/3	6/3/2/11	19/10/4/2
BMI (kg/m^2^)	30/3	13/9	22/13
OTUs	1749	1285	1696
Coverage	1.00	1.00	1.00
Diversity index (median)			
Species	465	466	487
ACE	552.31	556.20	590.50
CHAO1	542.56	561.19	578.41
Shannon	5.51	5.36	5.69
Simpson	0.93	0.90	0.94
Beta diversity	0.39	0.42	0.40

*NBNC-HCC* non-HBV non-HCV related hepatocellular carcinoma, *B-HCC* HBV related hepatocellular carcinoma. Drinking condition, none/low level/medium level/high level, *BMI* Body Mass Index (kg/cm^2^), normal/overweight, *OTU* operational taxonomic units, *ACE* abundance-based coverage estimators

### Alterations of gut microbiota composition in HCC patients

**Fig. 1:**
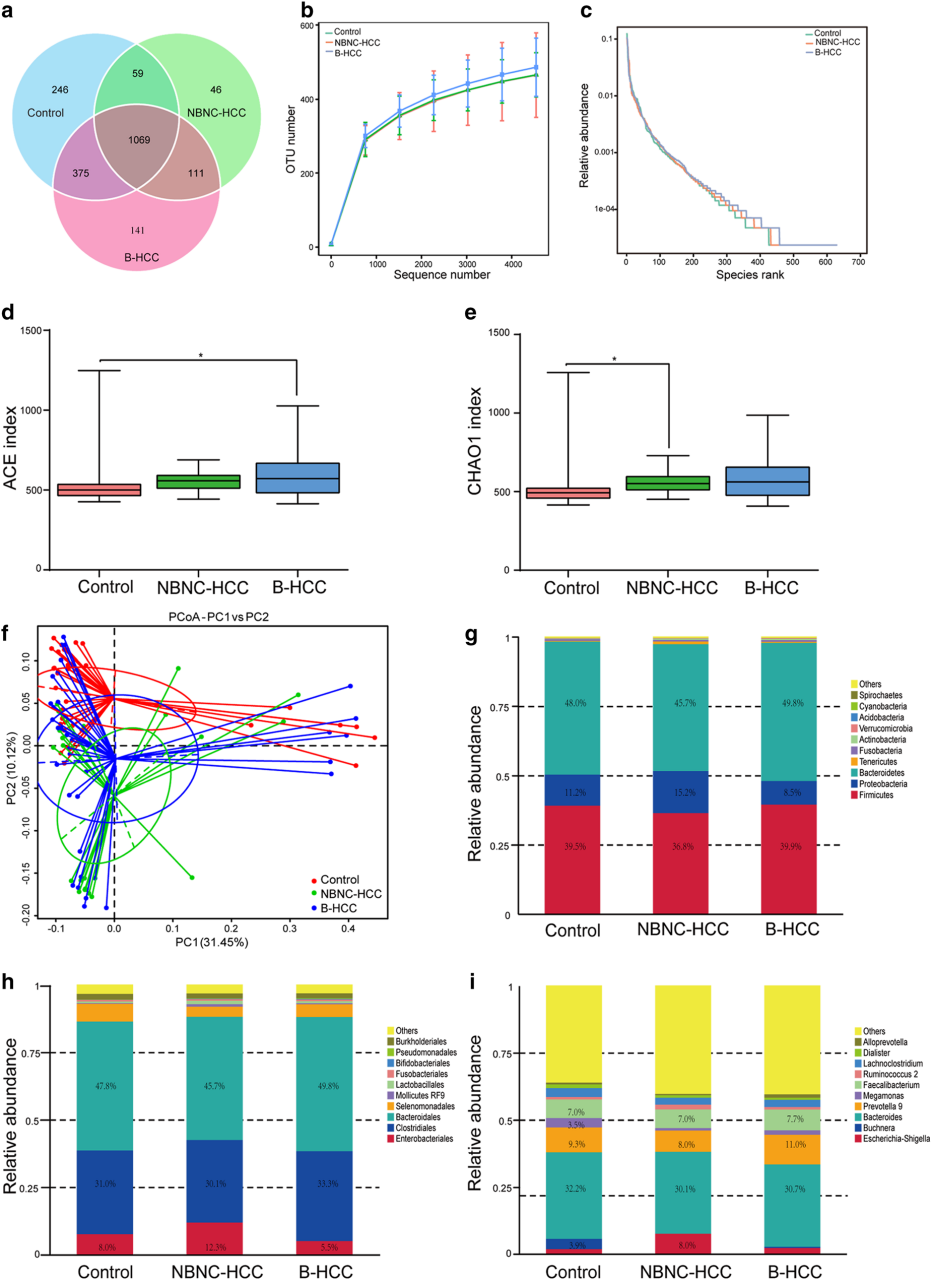
Shift microbiota and diversity in gut microbiota of healthy controls, NBNC-HCC and B-HCC patients. **a** Venn diagram of OTUs in three groups. **b** Observed species numbers in three groups. **c** Rarefaction curve for the comparison of OTUs in three groups. **d**, **e** Comparison of the alpha diversity (ACE and CHAO1) based on the OTUs profiles. **f** Principal Co-ordinates Analysis (PCoA) of bacterial beta diversity based on the unweighted UniFrac distances. Each node represents each sample. Control, NBNC-HCC and B-HCC subjects are colored in red, green and blue, respectively. **g**–**i** Relative abundance of the top 10 microbiota at the phylum, order and genus level

The overlapping OTU of three groups were shown in a Venn diagram (Fig. 1a). These data demonstrated that 246, 46 and 141 OTUs were existed independently in healthy controls, NBNC-HCC and B-HCC patients, respectively. Based on the OTUs analysis, the bacterial communities of B-HCC patients tended to be more heterogeneous, whereas those of the healthy controls and NBNC-HCC patients exhibited similar patterns (Fig. 1b). According to the rank-abundance curves, species richness of B-HCC patients was much higher than other two groups, and all the OTUs were evenly distributed (Fig. 1c).

Generally, the bacterial alpha diversity indices (such as abundance-based coverage estimators (ACE) and CHAO1 index) in B-HCC patients was the highest (Fig. 1d, e). However, the Shannon and Simpson indices were not significantly different among three groups (P > 0.050, Table 1). The Principal Co-ordinate Analysis (PCoA) of beta diversity calculated on the unweighted UniFrac distances was used for clustering 90 samples into three distinct enterotypes (Fig. 1f).

Distinct differences in bacterial composition were observed among three groups. The microbiome contained 30 phyla, 125 orders and 479 genera in all fecal samples. *Bacteroidetes*, *Firmicutes* and *Proteobacteria* were the most abundant taxonomic groups (Fig. 1g, Additional file 2). The relative abundance of *Proteobacteria* (15.2%) in NBNC-HCC patients was individually higher than that in other two groups, while the *Proteobacteria* (8.5%) in B-HCC patients was less. On the contrary, the relative abundance of Firmicutes (36.8%) in NBNC-HCC patients was the lowest among three groups. Based on the order level (Fig. 1h, Additional file 3), *Enterobacteriales*, *Clostridiales*, *Bacteroidales* and *Selenomonadales* were the most abundant taxonomic groups. For genus level (Fig. 1i, Additional file 4), *Escherichia*-*Shigella*, *Buchnera*, *Bacteroides*, *Prevotella*, *Megamonas* and *Faecalibacterium* were predominant bacteria. The relative abundance of *Escherichia*-*Shigella* was much higher in NBNC-HCC patient (8.0%), however, the abundance of *Buchnera* and *Megamonas* were much smaller in NBNC-HCC and B-HCC patients. In B-HCC patients, the abundance of *Prevotella* was much greater than other two groups. Meanwhile, healthy controls had more *Buchnera* species.

**Fig. 2:**
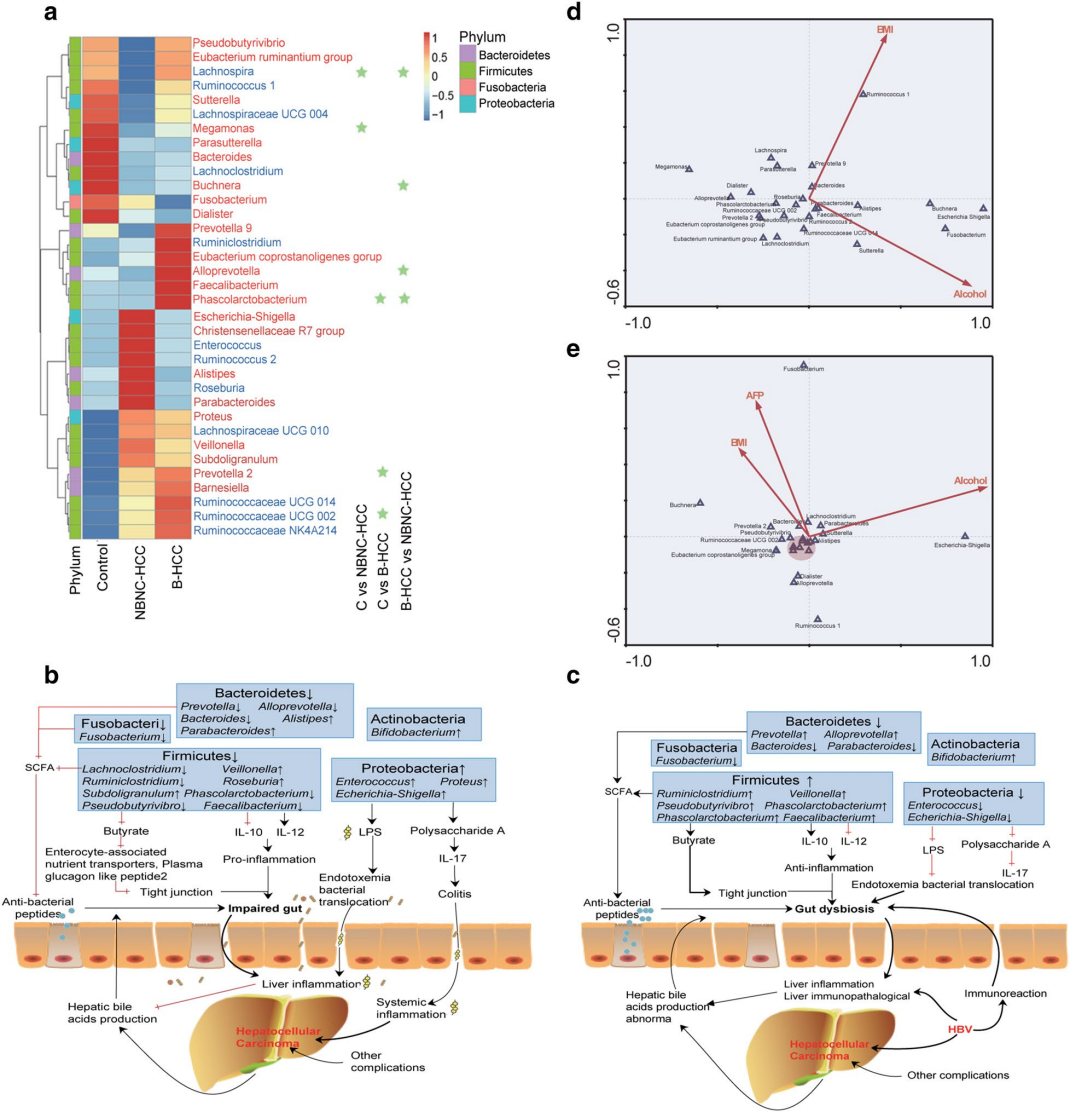
Different genera across three groups. **a** Heatmap of the top 35 genera. *P* < 0.050 by Wilcoxon rank sum test with light green star. Red in color represents Gram negative bacteria, blue in color represents Gram positive bacteria. **b** Changes in intestinal microbiota and the possible relations to intestinal dysfunction, gut dysbiosis and other complications in non-HBV non-HCV related HCC. **c** Changes in intestinal microbiota and the possible relations to intestinal dysfunction, gut dysbiosis and other complications in HBV related HCC. **d** The relationship between top 25 genera and body mass index (BMI) and alcohol. **e** The relationship between top 25 genera and body mass index (BMI), alcohol and alpha fetoprotein (AFP). The red circle shows the genera including *Lachnospira*, *Phascolarctobacterium*, *Ruminococcus* 2, *Parasutterella*, *Prevotella* 09, *Ruminococcaceae* UCG 014, *Eubacterium ruminantium* group, *Faecalibacterium*

Several similar findings were existed in the top 35 genera heatmap (Fig. 2a, Additional file 5), for example, *Proteus*, *Lachnospiraceae* UCG 010, *Veillonella*, *Subdoligranulum*, *Prevotella* 2, *Barnesiella* and *Ruminococcaceae* spp., were enriched both in NBNC-HCC and B-HCC patients. However, the differential abundance of bacteria have been found between NBNC-HCC and B-HCC patients displayed the reduced levels of *Faecalibacterium*, *Pseudobutyrivibrio*, *Lachnoclostridium*, *Ruminoclostridium*, *Prevotella* 9, *Alloprevotella* and *Phascolarctobacterium* (Fig. 2a, b), which may result in the decrease of potential anti-inflammatory short chain fatty acids (SCFAs), especially the butyrate [19, 20]. SCFAs which are intestinal microbial metabolites through dietary fiber play the anti-inflammatory effects on the immune systems [21–23]. Butyrate, the energy for enterocytes, which influences the intestinal barrier through the mucous production and tight junction [24]. In addition, *Faecalibacterium* inhibits interleukin (IL)-12 secretion and stimulates IL-10 [25] (Fig. 2b). In contrary, potential pro-inflammatory strains including *Escherichia*-*Shigella*, *Enterococcus*, *Proteus*, *Veillonella* increased in the gut of NBNC-HCC patients. For instance, *Enterococcus* can produce polysaccharide A and lipopolysaccharide (LPS), which suppressed IL-17 production, resulting in experimental colitis and promoted LPS translocated into the cell [26, 27].

Interestingly, a group of potential anti-inflammatory bacteria (such as *Prevotella*, *Alloprevotella*, *Faecalibacterium*, *Ruminiclostridium*) were increased in feces of B-HCC patients (Fig. 2a, c). It is well known that these bacteria are essential for healthy status. For instance, *Prevotella* is well known to produce propionate in healthy gut [28], and it may be play a protective effect in adult NAFLD patients [29]. Meanwhile, pro-inflammatory bacteria (such as *Escherichia*-*Shigella*, *Enterococcus*) were declined in fecal of B-HCC patients.

The Monte-Carlo tests of canonical correspondence analysis (CCA) revealed the top 25 genera were extremely influenced by alcohol (*P* = 0.017) and BMI (*P* = 0.007) (Fig. 2d, Additional file 6). For instance, *Escherichia*-*Shigella* was positively associated with alcohol factor. However, the *Ruminococcus* 2 was also positively associated with BMI factor. Alpha-fetoprotein (AFP) is one of the most useful markers for diagnosing and monitoring HCC [30]. In our study, AFP also had a strong influence on genus assemblages in HCC patients (*P* = 0.024, Fig. 2e). Due to the gut flora at different stages of liver disease various, so we also predicated the relationship between clinical data and the top 35 genera (Additional files 1 and 7). We calculated correlation of Spearman in all samples. The *P* value was corrected using the Holm method of R (Version 3.4.4, psych package). The clinical data were mainly focused on the common hepatic function index, which were alanine aminotransferase (ALT), aspartate aminotransferase (AST), glutamyl transpeptidase (GGT), total bilirubin (TBil), albumin and AFP. Several genera (e.g., *Enterococcus*, *Proteus*, *Tyzzerella* 4, *Parasutterella*, *Bifidobacterium*) were negatively correlated with GGT, ALT and AST, while *Dialister* were negatively correlated with albumin. In addition, the index TBil showed positive correction with *Parabacteroides*.

### Identification of potential bacteria biomarkers for HCC

**Fig. 3:**
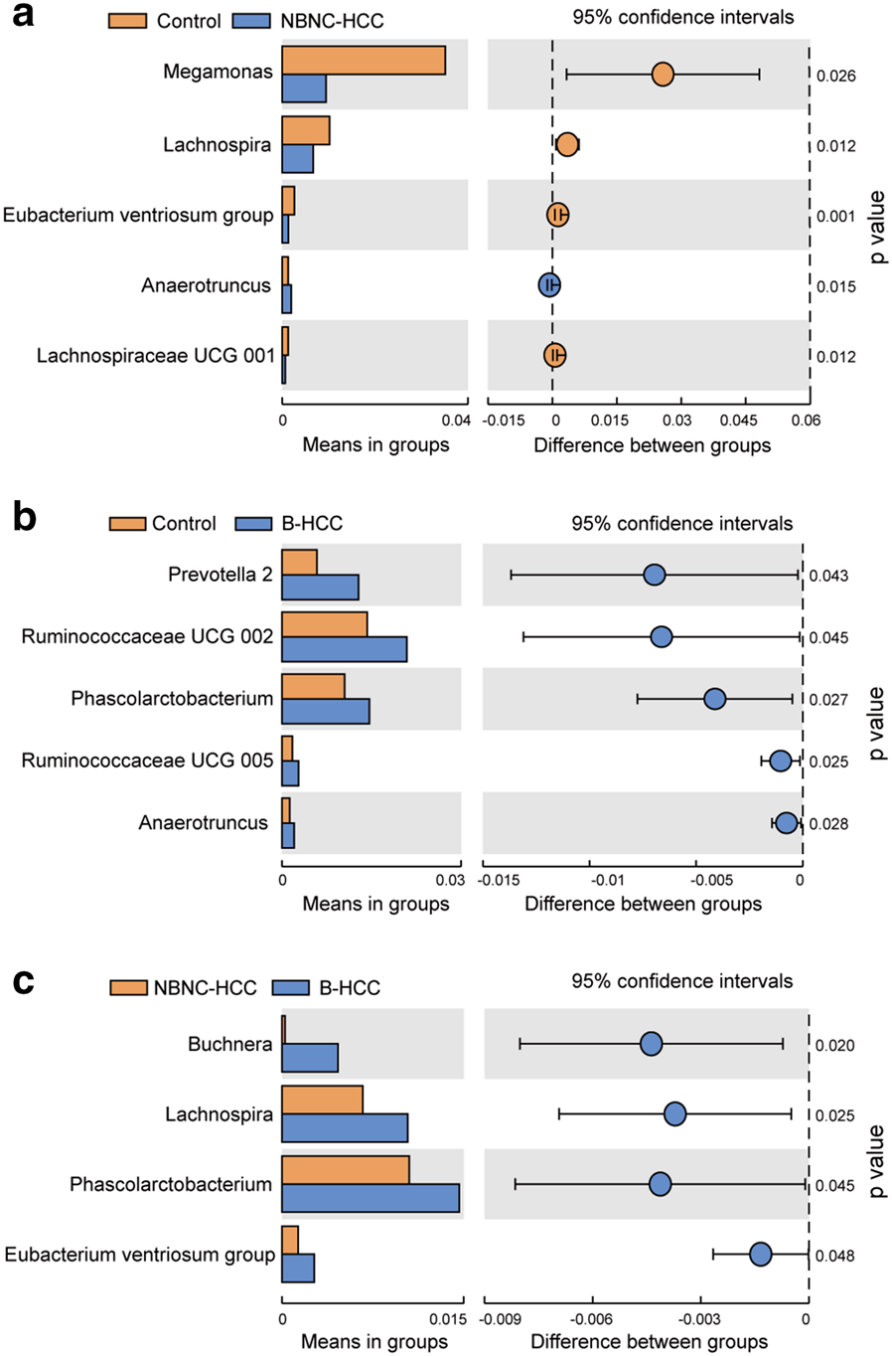
Genera strikingly different in gut microbiota of healthy controls (**a**), NBNC-HCC (**b**) and B-HCC (**c**)

At the genus level, *Megamonas*, *Lachnospira*, *Eubacterium ventriosum* and *Lachnospiraceae* UCG 001 were significantly decreased in NBNC-HCC patient samples as compared with healthy control samples (P < 0.050, Fig. 3a). In contrast, several genera such as *Prevotella*, *Phascolarctobacterium*, *Anaerotruncus* were particularly enriched in B-HCC patients than that in healthy controls (P < 0.050, Fig. 3b). The proportions of members of *Buchnera*, *Lachnospira*, *Phascolarctobacterium*, *Eubacterium ventriosum* were increased obviously in B-HCC patient samples compared with NBNC-HCC patients (Fig. 3c). Collectively, these differences revealed the dysbiosis involve in the development of HBV or non-HBV non-HCV related HCC.

Aberrant ecological networks of microbial communities occurred differently in B-HCC and NBNC-HCC patients.

**Fig. 4:**
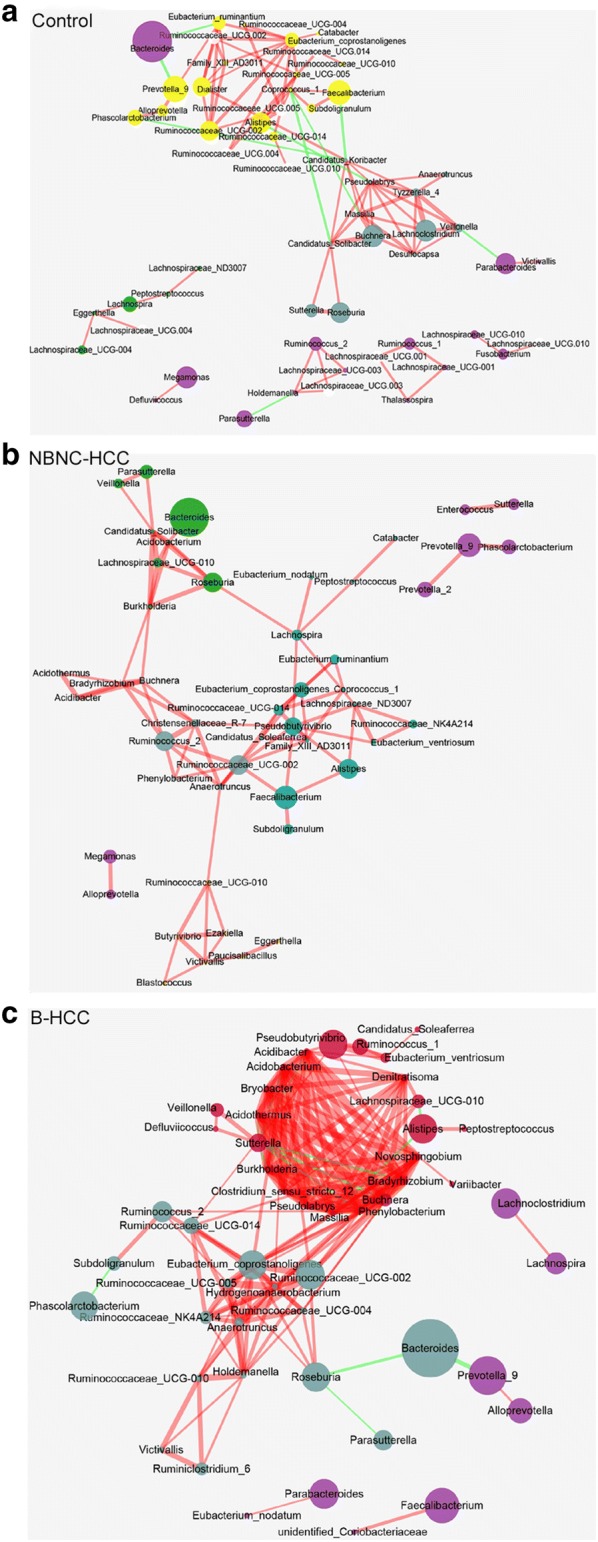
Networks to visualize interactions among different genera in three groups. **a** healthy controls; **b** NBNC-HCC patients; **c** B-HCC patients. Top 35 and significantly different genera are included. The density of the dashed line indicates the Pearson coefficient. Red links are the positive interactions between nodes, green links are the negative interactions. The size of node indicates the relative abundance

To explore the relationships among various genera (top 35 and the significantly different genera data, Additional files 5, 8), the ecological networks of three groups were visualized. A striking feature was that taxonomically related genera tend to cluster in healthy controls (Fig. 4a). It was obvious that the within network highly associated connections of healthy controls occupied significant position and the interactions between these nodes were major balance. In NBNC-HCC patients (Fig. 4b), perhaps due to the differences in diet and excessively drinking levels (72.73%, Table 1), NBNC-HCC patients displayed a simpler concurrent network with fewer integrated symbiosis compared to healthy controls. All the interactions of bacteria in NBNC-HCC patients were positively interactions. Nevertheless, patients with B-HCC displayed multifaceted network with lots of genera, and totally grouped into a solo module with association to many other modules (Fig. 4c). Most of the bacteria related to inflammation gathered together. In this small symbiotic network, most of the interactions showed a stronger positive relation, such as *Clostridium*, *Bryobacter*, *Lachnospiraceae*, *Buchnera*, *Burkholderia*, *Pseudobutyrivibrio*. However, fewer interactions were negative, such as *Alistipes*, *Bradyrhizobium* and *Sutterella*, which implicated in competitive relationships for different genera. These observations implicated that the intestinal ecosystem becomes permissive for the development and maintenance of the related taxa in HCC patients.

The potential multiple biological pathways of NBNC-HCC were different from other two groups.

**Fig. 5:**
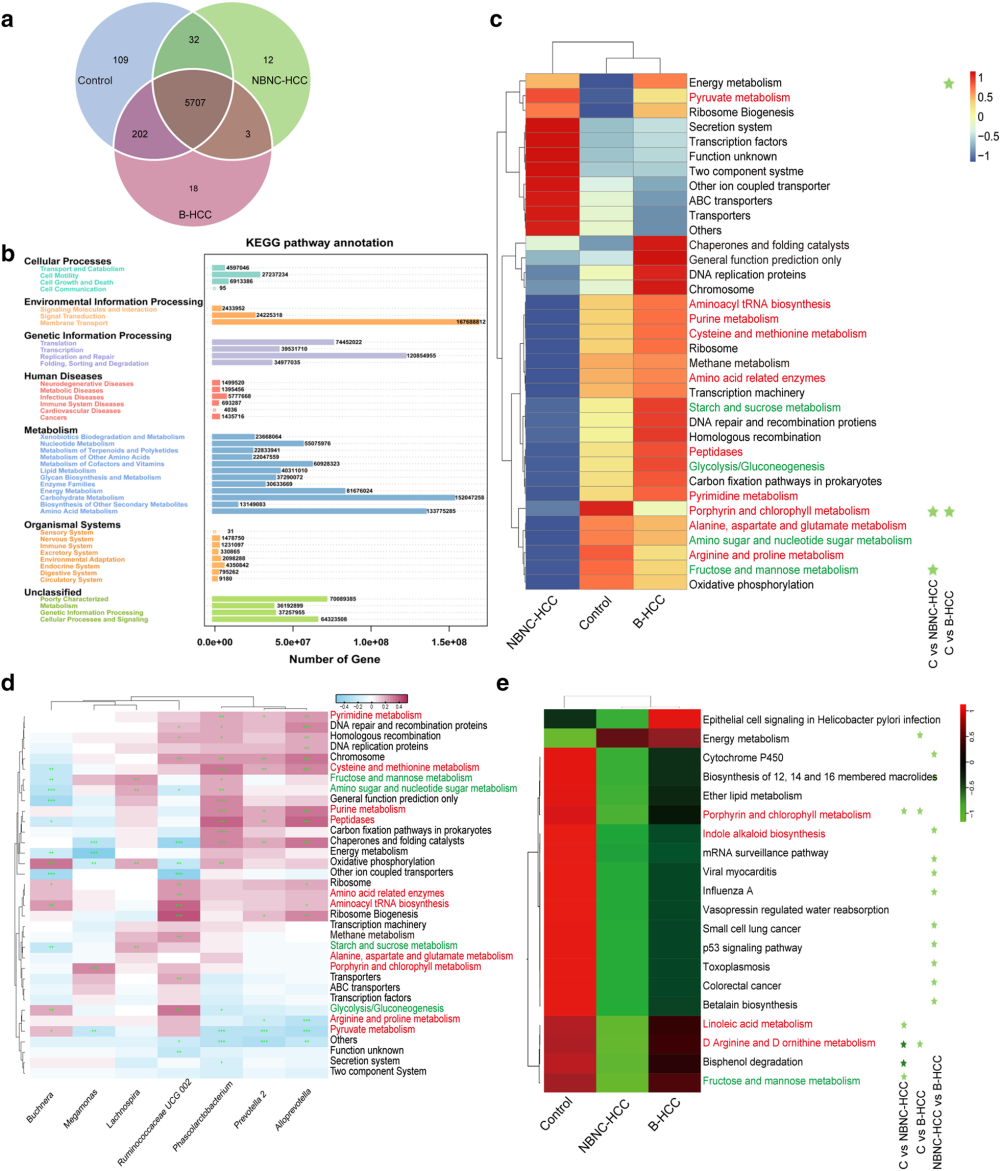
Predicated function and multiple biological pathways in three groups. **a** Venn diagram of the predicated multiple biological pathways. **b** Annotated of multiple biological pathway distribution in seven major categories. **c** Heatmap of the top 35 biological pathways across three groups. **d** The relationship between top 35 biological pathways and genera at the criteria of *P* < 0.050 by Wilcoxon rank sum test in top 35. ^+^*P* < 0.050; ^++^*P* < 0.010; ^+++^*P* < 0.001 by Spearman’s correlation analysis. **e** Heatmap of multiple biological pathways across three groups at the *P* < 0.100 by Wilcoxon rank sum test. Multiple biological pathways at *P* < 0.050 by Wilcoxon rank sum test are marked with light green star, *P* < 0.001 with dark star. Red in color relates with amino acid metabolism, green in color relates to glucose metabolism

To further understand the biological functions of genera among HCC patients and healthy controls, we performed Kyoto Encyclopedia of Genes and Genomes database (KEGG) analysis associated with gut microbiota [31]. We identified 15,039 biological pathways in all data. The predicated functions showed unique 109 for healthy controls, 12 for NBNC-HCC patients, and 18 for B-HCC patients (Fig. 5a). The mapped results indicated that multiple biological pathways were divided into seven branches (Fig. 5b). For instance, membrane transport, replication and repair, carbohydrate metabolism and amino acid metabolism were the predominant pathways.

Healthy controls and B-HCC patients displayed similar pathways regarding to the top 35 multiple biological pathways (Fig. 5c, Additional files 8, and 9). However, B-HCC patients showed higher abundance of pathways related to chaperones and folding catalysts, general function prediction, DNA replication proteins and chromosome, which further supported that the HBV can destroy the normal function of DNA [5]. In addition, NBNC-HCC patients showed lower abundance of pathways related to amino acid metabolism (such as purine, cysteine and methionine, in red color) and glucose metabolism (such as starch and sucrose, glycolysis/gluconeogenesis, fructose and mannose, in green color). Meanwhile, KEGG analysis showed that microbial functional genes involved in oxidative phosphorylation, amino sugar and nucleotide sugar metabolism were also declined in B-HCC patients. It was also reported that marked depletion of amino acids and nucleotides metabolism in alcohol related cirrhosis patients [32]. In agreement with the study, we noted that some types of transport such as secretion system, transcription factors, other in coupled transporter and ABC transporters are enrichment in the multiple biological pathways of NBNC-HCC patients.

The relationships of multiple biological pathways were predicated to be associated with seven significantly different genera of top 35 genera (Fig. 5d). For instance, *Phascolarctobacterium* and *Alloprevotella* involved in the similar potential pathways, such as pyrimidine metabolism, cysteine and methionine metabolism and peptidases. Both genera had a negative relationship to arginine and proline metabolism and pyruvate metabolism. In addition, *Ruminococcaceae* UCG 002 involved in the potential pathways related to transporter, ribosome, ribosome biogenesis, chromosome, amino acid metabolism (Fig. 5d). Meanwhile, *Lachnospira* was potentially associated with nucleotide sugar metabolism, amino sugar, fructose and mannose metabolism.

We also identified significant changes in the multiple biological pathways of three groups (Fig. 5e). Five significantly discriminative metabolic pathways (D Arginine and D ornithine metabolism, bisphenol degradation, porphyrin and chlorophyll metabolism, linoleic acid metabolism, fructose and mannose metabolism) between healthy controls and NBNC-HCC patients, and three significantly discriminative pathways (energy metabolism, porphyrin and chlorophyll metabolism, D Arginine and D ornithine metabolism) between healthy controls and B-HCC patients. Due to the taxonomic microbiome, composition was different between two HCC groups, 11 significantly discriminative pathways (biosynthesis of 12, 14 and 16 membered macrolides, mRNA surveillance pathway, indole alkaloid biosynthesis, p53 signaling pathway, small cell lung cancer, toxoplasmosis, betalain biosynthesis, influenza A, viral myocarditis, colorectal cancer, cytochrome P450) between NBNC-HCC and B-HCC patients. The metabolic pathways such as tetracycline biosynthesis and tyrosine metabolism showed divergence enrichment degree among three groups (Additional files 10, 11, 12, 13, 14, 15).

## Discussion

Our study focused on comparing the gut microbiota of HCC patients with HBV or without HBV/HCV infection. Like other bacteria associated with hepatic diseases, the bacterial diversity level and composition varied differently between NBNC-HCC and B-HCC patients. Generally, B-HCC patients were found to harbor higher species richness. At the phylum level, there was a decrease in *Firmicutes* and an increase in *Proteobacteria* of NBNC-HCC patients. However, *Proteobacteria* decreased in B-HCC patients. We found that NBNC-HCC patients harbored fewer potential anti-inflammatory bacteria and more pro-inflammatory bacteria. On the contrary, the B-HCC patients harbored more potential anti-inflammatory bacteria. Taken together, our analysis implicated that the gut microbiota plays an important role in the progression of HBV or non-HBV non-HCV related HCC.

It has been reported that *Lactobacillus* and *Bifidobacterium* were less in liver diseases [15, 33], but our study found both bacteria were increased in HCC patients and decreased in healthy controls (Additional file 4). Lactobacillus and *Bifidobacterium* were important probiotics to maintain intestinal microbial homeostasis and gut epithelial barrier [34]. However, recent studies reported that a higher abundance of *Bifidobacterium* were found be in the tissues of patients with colorectal adenomas or carcinoma [35, 36]. We hypothesized the amount of *Bifidobacterium* in colorectal adenomas or carcinoma tissue might be related with the tumor differentiation, mucosal barrier, and higher immune response to disease. For instance, *Bifidobacterium longum* nearly abolished melanoma tumor outgrowth by promoting anti-PD-L1 therapy [37]. By contrast, a recent report showed that commensal *Bifidobacterium pseudolongum* promoted the development of pancreatic cancer [38]. Thus, the different species of probiotics may have diverging effects in the tumor microenvironment. Taken together, the increasing abundance of *Lactobacillus* and *Bifidobacterium* in HCC patients remind us that it should be more attention paid on the probiotic function.

The NBNC-HCC patients harbored fewer anti-inflammatory bacteria and more pro-inflammatory bacteria in our study (Fig. 2a, b). We also noted the higher rate of alcohol consumption in NBNC-HCC patients (72.73%) (Table 1). Heavy drinking of alcohol causes inflammation of numerous organs. The alcohol correlated with pro-inflammatory bacteria, such as *Escherichia*-*Shigella* and *Enterococcus* would enhance leaky gut to the gut dysbiosis [39]. In addition, *Escherichia* overgrown to interfere the health equilibrium, which enters the liver through blood circulation, leading to the disorder of fatty acid metabolism [26], and becoming component of NAFLD pathogenesis [40]. In agreement with previous studies, our analysis further implicated that the diet and lifestyle habits play a vital role in the development of non-HBV non-HCV related HCC patients.

The B-HCC patients with more potential anti-inflammatory bacteria (such as *Prevotella*, *Faecalibacterium*) and fewer pro-inflammatory bacteria (such as *Escherichia*-*Shigella*, *Enterococcus*) were different from previous reports about HBV induced liver diseases [15, 41]. The discrepancy with our findings was probably the progression of liver diseases. The previous studies involved in HBV induced chronic carriers or liver cirrhosis, whereas the subjects of our study were from the HCC. There are essential differences between liver cirrhosis and HCC in accordance with pathogenesis, radiographic measurements, clinical symptoms and signs and other complications. Furthermore, immunoreaction was considered as a significant characteristic in the progression of HCC [3]. HBV uses multiple biological pathways to harness host innate immunity to enhance its replication, which initiates the immunological mechanisms to defense the acute or chronic infection [5]. Therefore, the discrepancy of fecal microbiota between the B-HCC and NBNC-HCC patients in our study perhaps ascribe to the HBV infection. The protective T-cell memory were lack in the chronic HBV infection, and T-cell responses also exhaustion [5, 42]. So, we infer the increased anti-inflammatory bacteria in B-HCC patients may be in response to HBV infection.

Interestingly, Ren et al. [18] indicated the butyrate-producing bacteria declined in early HCC patients, such as *Ruminococcus*, *Feacalibacterium*, *Clostridium*. However, the butyrate-producing bacteria presented heterogeneity in HBV and non-HBV non-HCV related HCC in our study. This further indicated HBV indeed play a role in changes of gut microbiota. Meanwhile, our study involved middle-aged adults (mean year is 56, Table 1, Additional file 1), whereas much older than the previous study [18]. In addition, 30 microbial markers were predicated using to identify early HCC in the previous study [18]. But some bacterial markers were not detected in the present study, such as *Gemmiger*. The conflict findings are possibly due to the individuals with different regions. The population of our study were all from Jiangsu Province. It has been reported that the diagnostic model of one location may be not used in other location, especially diagnostic efficiency declined with the geographic scale increased [43]. The characteristic changes of gut microbiota had a strongest relationship with the host location [43]. Thus, the diagnosis potential of microbial markers should be considered the geographic differences.

**Fig. 6:**
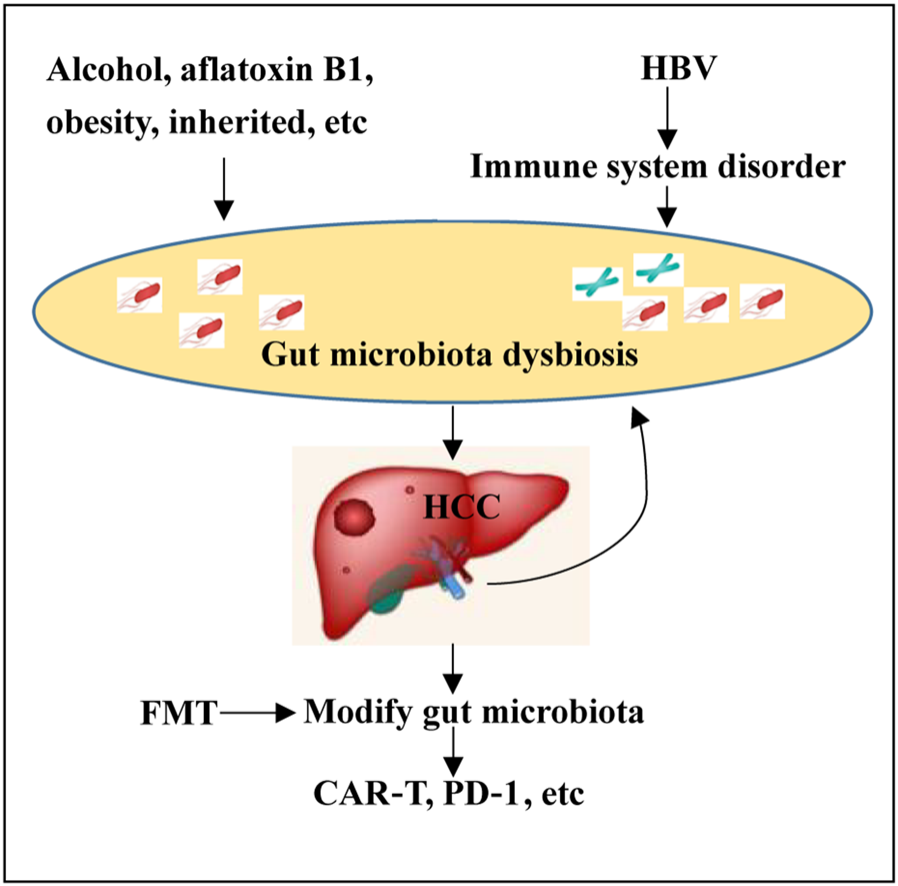
The predicated therapy of HCC patients. FMT, fecal microbiota transplantation; CAR-T, chimeric antigen receptor T-cells; PD-1, cell death protein-1

Currently, the treatment of HCC remains a challenge. Thus, it is necessary to develop an effective, life-prolonging strategy in the treatment of HCC patients. Recently, immunotherapy based on chimeric antigen receptor T-cell (CAR-T) [44, 45] or programmed cell death protein 1 (PD-1) [46, 47] has been proved as a promising strategy for cancer treatment. To date, the application of CAR-T cell therapy has some potential values in HCC [48]. In addition, fecal microbiota transplantation (FMT) can improve immune checkpoint inhibitors (ICI) associated colitis, which reconstructed the gut microbiome [49]. Based on the current microbiota analysis of HCC, we also suggest that the direct modification the gut microbiota of HCC patients associated with immunotherapy maybe beneficial for HCC patients (Fig. 6).

## Conclusions

Our study provides strong evidences that HBV and non-HBV HCV related HCC were associated with different bacteria and displayed the aberrant ecological networks of microbial communities. Our data also provided the additional evidences implicated that the different bacteria play a potential role in the tumorigenesis of both B-HCC and NBNC-HCC patients. We suggest that the gut-liver-axis can be used for monitoring and preventing the progression of liver disease and HCC.

## Methods

### Study subjects

A total of 57 HCC patients and 33 healthy controls who attended annual physical examination were recruited from September 2016 to May 2017 at Nanjing Medical University Affiliated Cancer Hospital. All participants were provided a written informed consent upon enrolment. This study was approved by the Ethics Committee of Nanjing Medical University.

HCC diagnosis is depending on three factors which includes chronic liver disease background, the positive iconography examination results, or the positive pathological examination. All HCC patients were free from other viral infections, such as Human Immunodeficiency Virus (HIV) [50]. These patients were also free from any other types of liver disease. The HCC patients underwent viral serologic testing (HBsAg and HCVAb). The HCC patients were separated into two groups. Based on the history of HBV or positive HBsAg for more than 6 months, the HBV infection was diagnosed. The patients with HBV were defined as HBV related HCC (B-HCC). While in the other group includes the patients without HBsAg or HCVAb, so-called non-HBV non-HCV related HCC (NBNC-HCC) [51]. The healthy controls were excluded diabetes, metabolic syndrome, hypertension, inflammatory bowel diseases, liver disease and cancers. All of them did not get any antiviral therapy or immunotherapy in the past 6 months.

The physiological characteristics of volunteers including age, weight and drinking condition were investigated (Table 1, Additional file 1). The standard of drinking history was considered as any alcoholic beverage (unit: gram) [52]. Men intake of alcohol less than 9.9 grams daily (or 4.9 g/days for women) was considered as low, while the consumption between 10 and 39.9 g/days (or women 5 and 19.9 g/days) was moderate, more than 40 g/days (or women 20 g/days) was high. Excepting only one HCC patients, 56 HCC patients had the blood test before enrolled, such as AFP, ALT, AST, GGT, TBil, albumin, serum creatinine (SCr) (Additional file 1). Further, the level of cirrhosis was evaluated according to the Child–Pugh and MELD [53, 54].

### Fecal sample collection, DNA extraction, PCR amplification, 16S rRNA sequencing

Fecal samples of each participant were obtained at hospital. The time span from sampling to Nanjing Medical University was intended within 24 h. Frozen samples were then stored at − 80 °C until analysis.

The genomic DNA of feces was extracted using kit (#DP328, Tiangen Biotech Co., Ltd., Beijing, China). The DNA concentration was detected using Qubit 2.0 Fluorometer (Thermo Fisher Scientific, USA). PCR was performed to produce V4 regions of the 16S rRNA gene using the conserved primers 515F (5′-GTGCCAGCMGCCGCGGTAA-3′) and 806R (5′-GGACTACHVGGGTWTCTAAT-3′), and no template DNA reaction was used as a negative control. PCR products were monitored using the 2% agarose gel. The strips between 400 and 450 bp were purified with GeneJET Gel Extraction Kit (Thermo Fisher Scientific, USA). PCR fragments were sequenced by Novogene Bioinformatics Technology Co., Ltd. (Tianjin, China).

## 16S rRNA data analysis

Illumina TruSeq DNA PCR-Free Library Preparation Kit (Illumina, USA) was used for generation of sequencing libraries. QIIME software 1.9 package was used to analyze sequences (Quantitative Insights Into Microbial Ecology, http://bio.cug.edu.cn/qiime/). Sequences having ≥ 97% resemblance were categorized as the same operational taxonomic units (OTUs). Alpha and beta diversity were calculated using the relative abundance of OTUs in each sample. The 16S rRNA data were assessed the potential multiple biological pathways of the gut microbiota using PICRUSt [55]. The KEGG ortholog identifiers (KO modules) were used to design the map of metabolic pathways in iPath 2. Datasets are publicly available (Accession number, GSE108847).

### Genera interaction in ecological networks of microbial community analysis

To elucidate genera interactions in each group, we constructed three groups of topological overlap networks. The topological overlap of OTU was clustered into modules using WGCNA package of R (Version 3.4.4). The network analysis was visualized using Cytoscape 3.5.1. The threshold was set by Pearson *r* > 0 *P* < 0.1, and the topology overlap > 0.01 [56]. The genera including the top 35 and the significantly different genera were used to do network analysis.

### Genera canonical correspondence analysis

To examine the distribution of genera associated with personal features (Body Mass Index, alcohol and AFP), canonical correspondence analysis (CCA) were visualized using the software CANOCO 4.5 [57]. Monte-Carlo permutation tests were performed to analyze the personal features played significant influence on the distribution of genera at *P* < 0.050. To avoid interference of rare species, top 25 genera were included.

### Statistical analysis

The alpha diversity index was analyzed suing the QIIME (Version 1.9). PCoA was performed using the ade4 package of R (Version 3.4.4). The comparison of bacterial taxonomic or KO modules was tested by Wilcoxon rank sum test, *P* value was corrected using the Benjamini–Hochberg method, which named as false discovery rate (FDR) value.

## Additional files

**Additional file 1.** Information of the study cohort used in this study. A total of 90 volunteers consisted of 33 healthy controls, 22 individuals of NBNC-HCC patients and 35 individuals of B-HCC patients were enrolled. The characteristics of participants including physical examination (gender, age, height, weight, BM, drinking condition), blood biochemical indexes (AFP, ALT, AST, GGT, TBil, INR, SCr, total protein, albumin, prothrombin time and activated partial thromboplastin time), Child–Pugh score and class, and MELD score.

**Additional file 2.** Relative abundance of bacteria at the phylum level.

**Additional file 3.** Relative abundance of bacteria at the order level.

**Additional file 4.** Relative abundance of bacteria at the genus level.

**Additional file 5.** Relative abundance of the top 35 genera.

**Additional file 6.** Relative abundance of the top 25 genera for canonical correspondence analysis.

**Additional file 7.** The relationship between six serologic indices (GST, AST, GGT, AFP, TBil, albumin) and top 35 genera is estimated by Spearman’s correlation analysis. *, *P* < 0.050; **, *P* < 0.010; ***, *P* < 0.001.

**Additional file 8.** Relative abundance of the significantly different genera (*P* < 0.050 by Wilcoxon rank sum test).

**Additional file 9.** Relative abundance of the top 35 predicated biological pathways.

**Additional file 10.** The differences in metabolic pathway between healthy controls and B-HCC patients. Green line is the special metabolism for healthy controls, yellow line is the special metabolism for B-HCC patients, red line for the common metabolism.

**Additional file 11.** The differences in metabolic pathway between healthy controls and NBNC-HCC patients. Green line is the special metabolism for healthy controls, yellow line is the special metabolism for NBNC-HCC patients, red line for the common metabolism.

**Additional file 12.** The differences in metabolic pathway between B-HCC and NBNC-HCC patients. Green line is the special metabolism for B-HCC patients, yellow line is the special metabolism for NBNC-HCC patients, red line for the common metabolism.

**Additional file 13.** The differences in biosynthesis and secondary metabolic pathway between healthy controls and B-HCC patients. Green line is the special metabolism for healthy controls, yellow line is the special metabolism for B-HCC patients, red line for the common metabolism.

**Additional file 14.** The differences in biosynthesis and secondary metabolic pathway between healthy controls and NBNC-HCC patients. Green line is the special metabolism for healthy controls, yellow line is the special metabolism for NBNC-HCC patients, red line for the common metabolism.

**Additional file 15.** The differences in biosynthesis and secondary metabolic pathway between B-HCC and NBNC-HCC patients. Green line is the special metabolism for B-HCC, yellow line is the special metabolism for NBNC-HCC patients, red line for the common metabolism.

## Abbreviations

AFP: alpha fetoprotein
ALT: alanine Aminotransferase
AST: aspartate aminotransferase
BMI: body mass index
CAR-T: chimeric antigen receptor T-cell
CCA: canonical correspondence analysis
FMT: fecal microbiota transplantation
GGT: glutamyl transpeptidase
B-HCC: HBV related hepatocellular carcinoma
HCC: hepatocellular carcinoma
INR: International Normalized Ratio
KEGG: Kyoto Encyclopedia of Genes and Genomes database
NAFLD: nonalcoholic fatty liver disease
NBNC-HCC: non-HBV non-HCV related hepatocellular carcinoma
PD-1: cell death protein-1
TBil: total bilirubin
