# Supplementary figures and images for: Alteration in gut microbiota associated with hepatitis B and non-hepatitis virus related hepatocellular carcinoma
Source: Gut Pathog. 2019 Jan 18;11:1. doi: 10.1186/s13099-018-0281-6 (PMC6337822; doi:10.1186/s13099-018-0281-6)

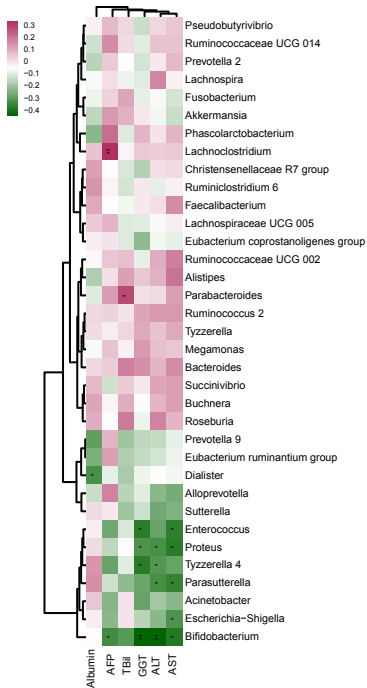

Supplement: Supplementary file 7 — Additional file 7. The relationship between six serologic indices (GST, AST, GGT, AFP, TBil, albumin) and top 35 genera is estimated by Spearman’s correlation analysis. *, P < 0.050; **, P < 0.010; ***, P < 0.001. [file 13099_2018_281_MOESM7_ESM.pdf]

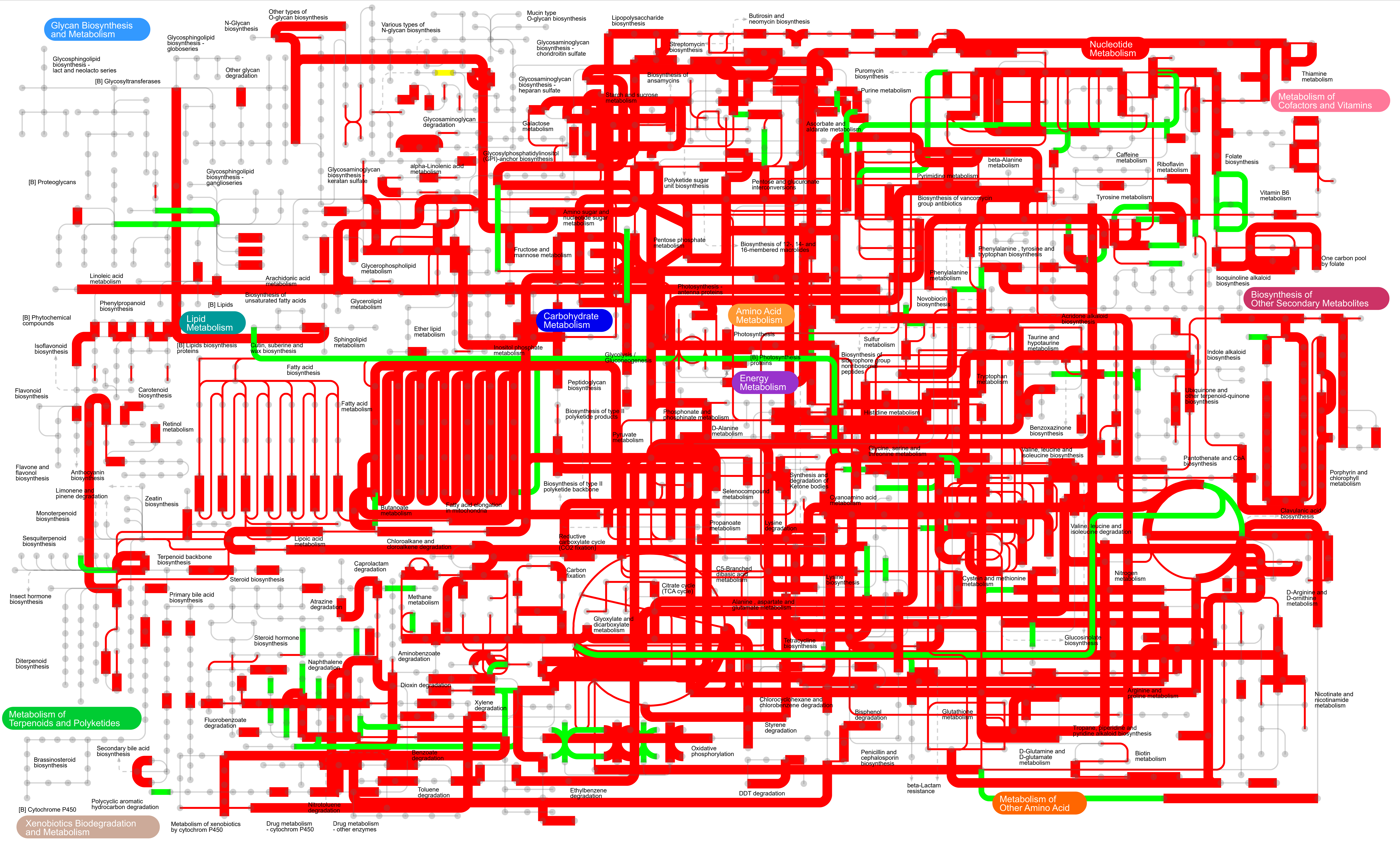

Supplement: Supplementary file 11 — Additional file 11. The differences in metabolic pathway between healthy controls and NBNC-HCC patients. Green line is the special metabolism for healthy controls, yellow line is the special metabolism for NBNC-HCC patients, red line for the common metabolism. [file 13099_2018_281_MOESM11_ESM.pdf]
