# Supplementary material for: Alteration in gut microbiota associated with hepatitis B and non-hepatitis virus related hepatocellular carcinoma
Source: Gut Pathog. 2019 Jan 18;11:1. doi: 10.1186/s13099-018-0281-6 (PMC6337822; doi:10.1186/s13099-018-0281-6)
Supplement: Supplementary file 13 — Additional file 13. The differences in biosynthesis and secondary metabolic pathway between healthy controls and B-HCC patients. Green line is the special metabolism for healthy controls, yellow line is the special metabolism for B-HCC patients, red line for the common metabolism. [file 13099_2018_281_MOESM13_ESM.pdf]

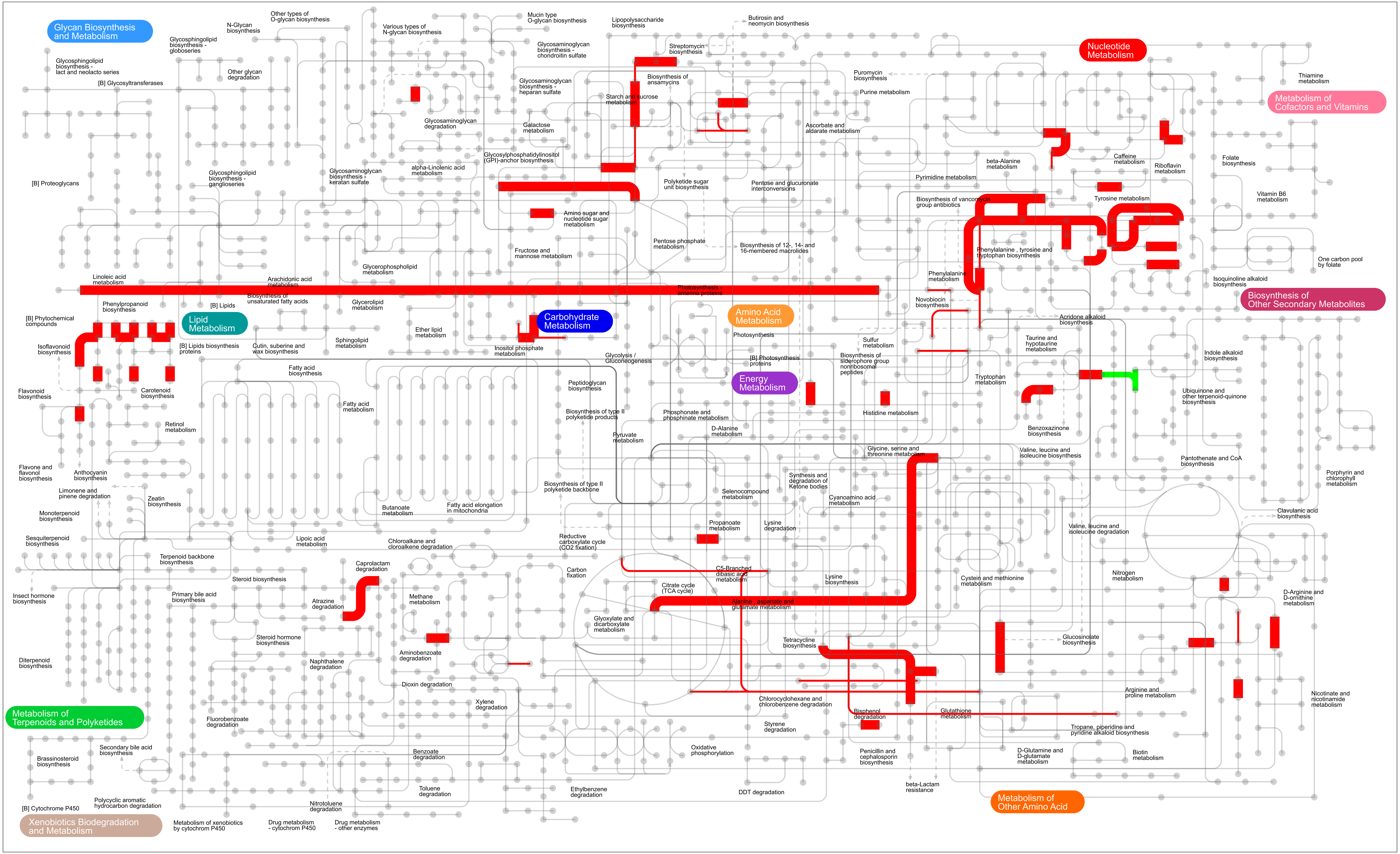
Glycan Biosynthesis and Metabolism
N-Glycan biosynthesis
Other types of O-glycan biosynthesis
Various types of N-glycan biosynthesis
Mucin type O-glycan biosynthesis
Lipopolysaccharide biosynthesis
Butirosin and neomycin biosynthesis
Streptomycin biosynthesis
Nucleotide Metabolism
Glycosphingolipid biosynthesis - globoseries
Glycosphingolipid biosynthesis - lact and neolacto series
Other glycan degradation
[B] Glycosyltransferases
Glycosaminoglycan biosynthesis - chondroitin sulfate
Biosynthesis of ansamycins
Puromycin biosynthesis
Thiamine metabolism
Purine metabolism
Metabolism of Cofactors and Vitamins
Glycosaminoglycan biosynthesis - heparan sulfate
Starch and sucrose metabolism
Glycosaminoglycan degradation
Galactose metabolism
Ascorbate and aldarate metabolism
Glycosylphosphatidylinositol (GPI)-anchor biosynthesis
Caffeine metabolism
Folate biosynthesis
beta-Alanine metabolism
Riboflavin metabolism
[B] Proteoglycans
Glycosphingolipid biosynthesis - ganglioseries
Glycosaminoglycan biosynthesis - keratan sulfate
alpha-Linolenic acid metabolism
Polyketide sugar unit biosynthesis
Pentose and glucuronate interconversions
Pyrimidine metabolism
Tyrosine metabolism
Vitamin B6 metabolism
Biosynthesis of vancomycin group antibiotics
Amino sugar and nucleotide sugar metabolism
Pentose phosphate metabolism
Biosynthesis of 12-, 14- and 16-membered macrolides
Phenylalanine , tyrosine and tryptophan biosynthesis
One carbon pool by folate
Fructose and mannose metabolism
Glycerophospholipid metabolism
Linoleic acid metabolism
Arachidonic acid metabolism
Photosynthesis - antenna proteins
Phenylalanine metabolism
Isoquinoline alkaloid biosynthesis
Biosynthesis of unsaturated fatty acids
Phenylpropanoid biosynthesis
[B] Lipids
Glycerolipid metabolism
Novobiocin biosynthesis
Biosynthesis of Other Secondary Metabolites
[B] Phytochemical compounds
Lipid Metabolism
Carbohydrate Metabolism
Amino Acid Metabolism
Acridone alkaloid biosynthesis
Ether lipid metabolism
Photosynthesis
Isoflavonoid biosynthesis
[B] Lipids biosynthesis proteins
Cutin, suberine and wax biosynthesis
Sphingolipid metabolism
Inositol phosphate metabolism
Sulfur metabolism
Taurine and hypotaurine metabolism
Glycolysis / Gluconeogenesis
[B] Photosynthesis proteins
Biosynthesis of siderophore group nonribosomal peptides
Indole alkaloid biosynthesis
Fatty acid biosynthesis
Energy Metabolism
Tryptophan metabolism
Flavonoid biosynthesis
Carotenoid biosynthesis
Peptidoglycan biosynthesis
Ubiquinone and other terpenoid-quinone biosynthesis
Fatty acid metabolism
Biosynthesis of type II polyketide products
Phosphonate and phosphinate metabolism
Histidine metabolism
Benzoxazinone biosynthesis
Retinol metabolism
D-Alanine metabolism
Pyruvate metabolism
Glycine, serine and threonine metabolism
Valine, leucine and isoleucine biosynthesis
Pantothenate and CoA biosynthesis
Flavone and flavonol biosynthesis
Anthocyanin biosynthesis
Synthesis and degradation of Ketone bodies
Porphyrin and chlorophyll metabolism
Limonene and pinene degradation
Biosynthesis of type II polyketide backbone
Selenocompound metabolism
Cyanoamino acid metabolism
Zeatin biosynthesis
Butanoate metabolism
Fatty acid elongation in mitochondria
Monoterpenoid biosynthesis
Clavulanic acid biosynthesis
Propanoate metabolism
Lysine degradation
Valine, leucine and isoleucine degradation
Sesquiterpenoid biosynthesis
Lipoic acid metabolism
Chloroalkane and cloroalkene degradation
Reductive carboxylate cycle (CO2 fixation)
Terpenoid backbone biosynthesis
Caprolactam degradation
Carbon fixation
C5-Branched dibasic acid metabolism
Nitrogen metabolism
Steroid biosynthesis
Lysine biosynthesis
Cystein and methionine metabolism
Citrate cycle (TCA cycle)
D-Arginine and D-ornithine metabolism
Insect hormone biosynthesis
Primary bile acid biosynthesis
Atrazine degradation
Methane metabolism
Alanine , aspartate and glutamate metabolism
Glyoxylate and dicarboxylate metabolism
Steroid hormone biosynthesis
Aminobenzoate degradation
Tetracycline biosynthesis
Glucosinolate biosynthesis
Diterpenoid biosynthesis
Naphthalene degradation
Dioxin degradation
Arginine and proline metabolism
Xylene degradation
Chlorocyclohexane and chlorobenzene degradation
Nicotinate and nicotinamide metabolism
Bisphenol degradation
Glutathione metabolism
Metabolism of Terpenoids and Polyketides
Fluorobenzoate degradation
Styrene degradation
Tropane, piperidine and pyridine alkaloid biosynthesis
Secondary bile acid biosynthesis
Oxidative phosphorylation
Brassinosteroid biosynthesis
Benzoate degradation
Penicillin and cephalosporin biosynthesis
D-Glutamine and D-glutamate metabolism
Biotin metabolism
beta-Lactam resistance
Polycyclic aromatic hydrocarbon degradation
Toluene degradation
Ethylbenzene degradation
DDT degradation
Metabolism of Other Amino Acid
[B] Cytochrome P450
Nitrotoluene degradation
Xenobiotics Biodegradation and Metabolism
Metabolism of xenobiotics by cytochrome P450
Drug metabolism - cytochrome P450
Drug metabolism - other enzymes
